# Ethnic Differences of Urinary Cadmium in Cigarette Smokers from the Multiethnic Cohort Study

**DOI:** 10.3390/ijerph18052669

**Published:** 2021-03-06

**Authors:** Shannon S. Cigan, Sharon E. Murphy, Bruce H. Alexander, Daniel O. Stram, Dorothy K. Hatsukami, Loic Le Marchand, Sungshim L. Park, Irina Stepanov

**Affiliations:** 1 Department of Pediatrics, Division of Epidemiology and Clinical Research, University of Minnesota, Minneapolis, MN 55455, USA; 2 Division of Environmental Health Sciences, School of Public Health, University of Minnesota, Minneapolis, MN 55455, USA; bruce.alexander@colostate.edu (B.H.A.); stepa011@umn.edu (I.S.); 3 Department of Biochemistry Molecular Biology and Physics, University of Minnesota, Minneapolis, MN 55455, USA; murph062@umn.edu; 4 Masonic Cancer Center, University of Minnesota, Minneapolis, MN 55455, USA; hatsu001@umn.edu; 5 Department of Environmental and Radiological Health Sciences, Colorado State University, Fort Collins, CO 80523, USA; 6 Department of Preventative Medicine, Keck School of Medicine, University of Southern California, Los Angeles, CA 90033, USA; Daniel.Stram@med.usc.edu; 7 Department of Psychiatry, University of Minnesota Medical School, Minneapolis, MN 55455, USA; 8 Epidemiology Program, University of Hawaii Cancer Center, Honolulu, HI 96813, USA; Loic@cc.hawaii.edu (L.L.M.); lpark@cc.hawaii.edu (S.L.P.)

**Keywords:** urinary cadmium, biomarkers, cigarette smoking, cadmium exposure, occupational exposures

## Abstract

The Multiethnic Cohort Study (MEC) has demonstrated racial/ethnic differences in smoking-associated lung cancer risk. As part of the ongoing effort to characterize exposure to cigarette smoke constituents and better understand risk differences, we evaluated Cd exposure as it is a known lung carcinogen. We quantified urinary cadmium (Cd) by inductively coupled plasma mass spectrometry in a subset of 1956 current smokers from MEC. Ethnic-specific geometric means (GM) were compared adjusting for age at urine collection, sex, creatinine (natural log), education, and smoking (urinary total nicotine equivalents [TNE] and smoking duration). Self-reported questionnaire data, including occupation, were also considered. Latinos and Native Hawaiians had the highest GM urinary Cd (0.871 and 0.836 ng/mL, respectively) followed by Japanese Americans and African Americans (0.811 ng/mL and 0.807, respectively) and Whites (0.736 ng/mL). Patterns in race/ethnicity were consistent by sex such that females had the highest GM urinary Cd. When further adjusting for categorical occupational Cd exposure, racial/ethnic differences of Cd remained (*p* = 0.009). Findings suggest differences in urinary Cd among smokers across different racial/ethnic groups exist and highlight the importance in considering environmental sources of Cd exposure beyond smoking. These finding lay ground for future studies of individual characteristics that are associated with lower risk for cancer despite higher carcinogenic exposures.

## 1. Introduction

Research based on the Multiethnic Cohort Study (MEC) demonstrated important racial/ethnic differences in smoking-related lung cancer risk. For example, at similar levels of smoking, Native Hawaiian and African American smokers have a higher risk of lung cancer compared to Whites, whereas Japanese American and Latino smokers have a lower risk [1]. Biomarker-based assessment of exposure to quantify smoking dose and the uptake of specific tobacco toxicants and carcinogens is a key tool in examining the mechanistic underpinnings of the observed smoking-associated risk differences across diverse populations. Previously published and ongoing research on smokers from the MEC employ such biomarker-based approaches with the aim to comprehensively compare tobacco carcinogen exposures and effects across racial/ethnic groups [2,3,4,5,6,7]. Our study adds to this research effort by analyzing the levels of urinary cadmium (Cd) in a subset of MEC smokers.

Cd is a constituent of tobacco and cigarette smoke [8,9,10]. Based on human studies and sufficient evidence in animals, Cd has been classified as a Group 1 known human lung carcinogen by the International Agency for Research on Cancer (IARC) [11,12]. Long-term exposure to Cd can be measured via urinary Cd and has been shown to accurately reflect the amount of Cd in the body [13,14,15]. As such, multiple reports of urinary Cd in smokers and nonsmokers have consistently demonstrated an almost 2-fold higher geometric mean urinary Cd in current smokers than in nonsmokers indicating that smoking is a major source of Cd exposure [16,17,18]. Even among smokers there are reported inter-individual differences in urinary Cd which could be a result of differences in smoking dose which is influenced by nicotine metabolism [19]. Additionally, various studies suggest that urinary Cd levels differ by race/ethnicity, but no study has explored racial/ethnic differences exclusively among smokers [16,17,18].

In addition to smoking, certain occupations and industries can be a major source of Cd exposure via inhalation among smokers as Cd is also an environmental and industrial pollutant [11]. Recent studies using population-level data have shown that among the U.S. working population, a noteworthy number of workers in industries such as repair service, metal, mining, and transportation have urinary Cd levels 10- to 50-fold higher than current limits set by occupational standards and guidelines [20,21]. In fact, the IARC classification of Cd as a human lung carcinogen relied on occupational studies assessing human epidemiological evidence on such classification. However, such studies were primarily conducted in predominately White males and lacked information on smoking [11,14]. Lastly, trace amounts of Cd can also be found in certain foods and drinking water and could serve as an additional source of exposure to Cd. However, in smokers, the contribution of such sources to the overall Cd exposure is relatively minor compared to smoking and is not likely to play a key role in lung cancer risk [8].

Therefore, we aimed to analyze urinary Cd in current smokers at the time of urine collection from five different race/ethnicity groups from the MEC [1,22]. In addition, to address the growing concerns about the health effects of environmental Cd exposure and the reported variability of burden by race/ethnicity, we leveraged available self-reported occupation data to investigate the potential association of urinary Cd levels with relevant occupational categories after accounting for internal nicotine dose and self-reported smoking duration.

## 2. Materials and Methods

### 2.1. Study Population

Details of the MEC have been published previously [23]. Briefly, participants were recruited from Hawaii and California (primarily Los Angeles County) between 1993 and 1996. The cohort consists of 215,251 men and women aged 45 to 75 years at recruitment, primarily belonging to five self-reported racial/ethnic groups (African American, Japanese American, Latino, Native Hawaiian and White). Ten years after cohort entry, a randomly selected subset of MEC participants (approximately 70,000) provided a blood sample and an overnight urine sample (Hawaii) or first morning void urine sample (California). In addition, participants completed questionnaires that included average daily cigarette smoking during the past two weeks, smoking duration, and medication records. In this analysis, we characterize a randomly selected subset of the MEC biospecimen cohort who were current smokers at the time of biospecimen collection, lung cancer-free, willing to provide a urine and blood sample, and had their urine analyzed previously for biomarkers of internal nicotine dose (TNE), which reflects smoking intake as well as other metabolites of tobacco carcinogens [2,5]. For this specific analysis, we measured urinary Cd on 1977 healthy participants. Twenty-one participants were excluded from the analyses due to missing education and/or smoking duration.

The MEC study protocol was approved by the Institutional Review Boards at the University of Southern California (IRB Study #HS-16-00719), University of Hawaii (IRB Study #0912M75654) and the secondary data analysis reported here was approved by the University of Minnesota (IRB Study #00003366). Study participants provided written consent.

### 2.2. Analysis of Urinary Cadmium (Cd)

Urine samples were prepared by diluting 50 µL of urine with 250 µL of 2% nitric acid (trace-metal grade). The measurement of Cd in prepared urine samples was carried out by inductively coupled plasma mass spectrometry (ICP-MS) at the Wisconsin State Laboratory of Hygiene (WSLH) University of Wisconsin, Madison, which is certified for the analysis of Cd in biological and environmental samples [24]. The average of the three replicate readings was used in our analysis. The analysts were blinded to the origin of all urine samples. Quality control measures were incorporated at different stages to monitor analytical accuracy and ensure data validity and included: (i) multiple replicates of randomly selected urine samples blindly inserted throughout the sample set; (ii) negative control (2% nitric acid method blanks) and positive control (urine with known concentrations of Cd) samples prepared by the University of Minnesota (UMN) laboratory and added to the set; and (iii) WSLH instrument performance controls were included with each batch of samples. The method limit of quantification (LOQ) was 0.02 ng/mL Cd.

### 2.3. Creatinine and Nicotine Intake Biomarkers

Urinary creatinine was quantified using a colorimetric microplate assay (CRE34-K01) from Eagle Bioscience (Amherst, NH, USA). Urinary total nicotine equivalents (TNE), which represents the sum of nicotine and its metabolites in urine (nicotine, cotinine, trans-3′-hydroxycotinine, nicotine N-oxide, and corresponding glucuronide conjugates), was quantified previously by liquid chromatography-tandem mass spectrometry (LC-MS/MS) [2].

### 2.4. Occupational Cd Exposure Categories

Occupational Cd exposure was captured on the MEC baseline questionnaire through self-report via two questions regarding longest occupational category worked and history of industry and occupations employed for more than 10 years [25]. A categorical variable was defined, a priori, based on a comprehensive literature review of occupations and industries reported to be associated with Cd exposure [8,11,21,26]. Based on the participants’ combined responses pertaining to the type of industry and occupation they maintained the longest, participants were grouped into four categories of occupational Cd exposure (Supplemental Table S1): “Likely exposed” (both the industry and occupation reported by a participant was known to be a source of Cd exposure), “Possibly exposed” (only one of the reported industry or occupation were a known source of Cd exposure); “Not likely exposed” (neither industry or occupation was a known source of Cd exposure) and “Unknown exposure” (participants who selected other or none for the offered response options or did not report).

### 2.5. Statistical Analysis

Urinary biomarkers of Cd and creatinine were log-transformed using the natural logarithm to approximate a normal distribution. Cd values below the LOQ were left-censored using appropriate methods [26]. To examine urinary Cd across racial/ethnic groups, a censored multiple linear regression model (tobit regression) was used to compute covariate-adjusted geometric means with estimated 95% confidence intervals (CI) to characterize precision. The base model was adjusted for age at urine collection, sex, creatinine (natural log) and education level (≤12th grade, vocational school/some college or ≥graduated college). Models were also adjusted for TNE and average smoking duration at time of urine collection (years). Smoking duration was assessed by self-report from baseline questionnaires at time of urine collection. Missing variables were imputed as previously reported (2). We considered additional variables as potential confounders, including CYP2A6 activity (a measure of nicotine metabolism), body-mass index (categories: underweight [<18.5 kg/m^2^], normal weight [18.5–24.9 kg/m^2^], overweight [25–29.9 kg/m^2^], and obesity [≥30 kg/m^2^] and medication use at time of urine collection as this may affect urine output (drug class [yes/no]: antidiabetic, antihypertension diuretic and specifically, hydrochlorothiazide/dyazide/lasix medication use) but since they were not significantly associated with urinary Cd levels in this cohort, we did not include them in the final model. To evaluate the relationship between urinary Cd levels and occupational Cd exposure, independent of smoking, models were adjusted for age at urine collection, sex, race/ethnicity, creatinine (natural log), education level, and smoking (TNE and smoking duration).

All geometric mean values and 95% CIs were estimated and back transformed to the original scale using the beta coefficients from the models and including a function of the variance of the errors to produce unbiased estimates. White was used as the referent in all racial/ethnic analysis as this group had the lowest mean level of urinary Cd. Chi-squared test, and Kruskal–Wallis tests were used, where appropriate. The interaction between race/ethnicity and occupational Cd exposure categories was assessed and retained if *p* < 0.05. All analyses were performed using Stata-IC statistical software (version 14; StataCorp LLC, College Station, TX, USA).

## 3. Results

Demographic and smoking (self-reported and biomarkers) characteristics of the study population overall and stratified by race/ethnicity are summarized in Table 1. A total of 1956 smokers between the ages of 46 to 87 years old at urine collection were included. Median years of smoking duration was highest in Whites (44.5 years) followed by Japanese Americans and Latinos (both groups 43.5 years) and lowest in both African Americans and Native Hawaiians (both groups 37.5 years). Median number of cigarettes per day (CPD) was highest among Whites (20 CPD), followed by Native Hawaiians (15 CPD), Japanese Americans (12 CPD), African Americans (10 CPD) and Latinos (8 CPD). Despite having one of the lowest reported median CPD, median TNE levels were highest in African Americans (44.5 nmol/mL), followed by Whites (35.7 nmol/mL), Latinos (32.7 nmol/mL), Native Hawaiians (30.3 nmol/mL) and lowest in Japanese Americans (27.4 nmol/mL).

**Figure 1:**
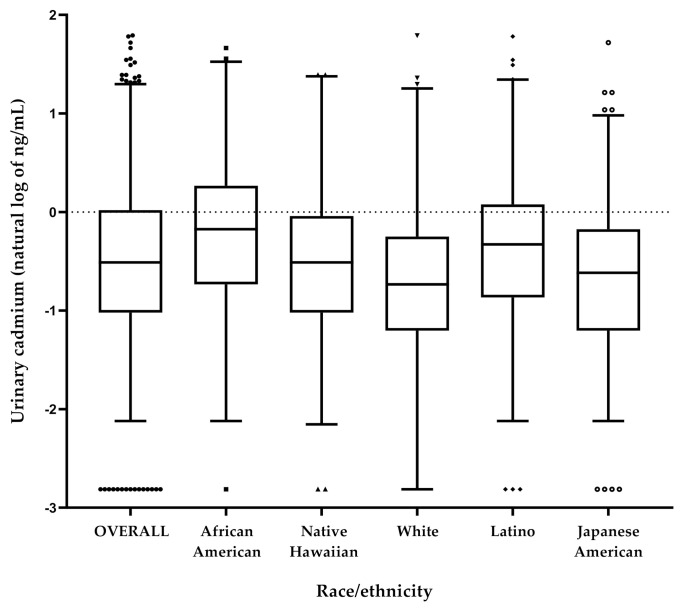
Median levels of urinary Cd (natural log of ng/mL) in MEC current smokers, overall and by race/ethnicity. The box represents the interquartile range (25th and 75th percentile), the dark line across the box represents the median value (50th percentile), the bottom and top whisker represents the first and 99th percentile and the circles above and below the whiskers represent outliers (>1.5× and <3× the interquartile range).

The overall median concentration of Cd in urine was 0.60 ng/mL and ranged from below the LOQ to 6.0 ng/mL (Figure 1 and .Supplemental Table S2). African Americans had the highest median urinary Cd levels (0.84 ng/mL), followed by Latinos (0.72 ng/mL), Native Hawaiians (0.60 ng/mL), Japanese Americans (0.54 ng/mL) and Whites (0.48 ng/mL). These patterns across race/ethnicity were consistent by sex such that median urinary Cd was higher in males in each racial/ethnic group. Urinary Cd was highly correlated with urinary TNE (r = 0.51; *p* < 0.001) and only weakly correlated with smoking duration (r = 0.07; *p* = 0.001). The ethnic-specific correlations between urinary Cd and urinary TNE ranged from r = 0.41 (Native Hawaiians) to r = 0.53 (African Americans).

### 3.1. Multivariate Analysis Adjusting for Smoking

After adjusting for race/ethnicity, age at urine collection, creatinine (natural log), and education level, the geometric mean urinary Cd levels were higher in females (0.880 ng/mL) compared to males (0.736 ng/mL; *p* < 0.001; Table 2). This difference remained after further adjusting for TNE and smoking duration (Model 2). Racial/ethnic specific analyses demonstrated urinary Cd levels were highest in Latinos and African Americans (0.834 and 0.821 ng/mL, respectively) followed by Native Hawaiians (0.815 ng/mL), Japanese Americans (0.781 ng/mL), and Whites (0.764 ng/mL) after adjusting for sex, age at urine collection, creatinine (natural log), and education level. Further adjustment for TNE and smoking duration (Model 2) resulted in the highest geometric mean levels of urinary Cd in Latinos (0.871 ng/mL) followed by Native Hawaiians (0.836 ng/mL), Japanese Americans (0.811 ng/mL), African Americans (0.807 ng/mL) and Whites (0.736 ng/mL). As previously reported in MEC smokers, African American smokers have significantly higher creatinine levels than Whites [4]. Therefore, an additional analyses dropping the adjustment for creatinine (adjusting only for sex, age at urine collection, education level, TNE and smoking duration) was evaluated and showed urinary Cd levels remained highest in Latinos (0.960 ng/mL) followed by African Americans (0.922 ng/mL), Native Hawaiians (0.844 ng/mL), Japanese Americans (0.796 ng/mL) and lowest in Whites (0.671 ng/mL; Supplemental Table S3). As a sensitivity analysis, TNE was log-transformed and modeled as TNE*smoking duration to represent cumulative TNE exposure (Supplemental Table S4), but it did not change the estimated geometric means within each racial/ethnic group substantially and therefore was not modeled this way in the final model for ease of interpretation.

### 3.2. Investigation of the Impact of Occupational Cd Exposure Categories

Analyses of urinary Cd levels by occupational Cd exposure categories demonstrated that, participants grouped in the ‘Likely exposed’ occupational Cd exposure category had 18.6% higher geometric mean urinary Cd compared to the ‘Not Likely exposed’ category (0.924 ng/mL versus 0.779 ng/mL; *p* = 0.002, Table 2) after adjustment for race/ethnicity, sex, age at urine collection, creatinine (natural log), and education level. The geometric mean in the “Possibly exposed” category was 8.7% higher than those in the “Not Likely exposed” category (0.847 ng/mL; *p* = 0.034). Participants with “Unknown exposure” had the lowest geometric mean urinary Cd level (0.775 ng/mL) but this did not differ from the “Not Likely exposed” category (*p* = 0.885). When further adjusted for TNE and smoking duration, results were in the same direction, but the level of significance was attenuated. 

**Figure 2:**
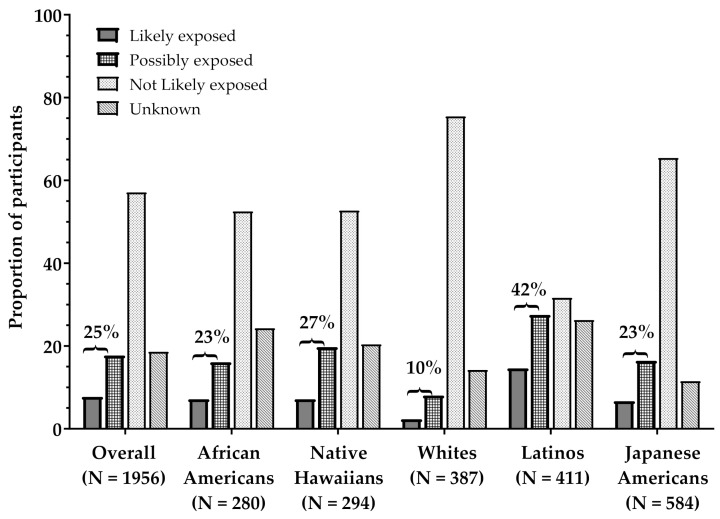
Proportion of Multiethnic Cohort (MEC) current smokers overall and within each racial/ethnic group assigned to occupational Cd exposure categories. Bracketed percentages represent the sum of Likely exposed and Possibly exposed in each group.

**Figure 3:**
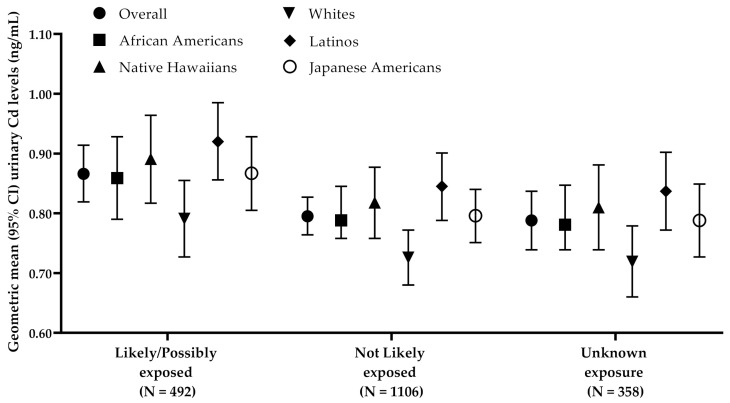
Geometric mean (95% CI) of urinary cadmium concentrations (ng/mL) by occupational cadmium exposure categories, presented overall and by race/ethnicity. Geometric means were adjusted for self-reported race/ethnicity (for overall specific model), sex, age at urine collection, creatinine (natural log), maximum education attainment level, urinary total nicotine equivalents (TNE), and smoking duration.

The relative proportion of participants within each occupational Cd exposure category differed by race/ethnicity (*p* < 0.001; Figure 2). Notably, a greater proportion of Latinos (42%) and Native Hawaiians (27%) were in the “Likely exposed” and “Possibly exposed” occupational Cd exposure categories. A greater proportion of all Whites (76%) and Japanese Americans (65%) were in the “Not Likely exposed” occupational Cd exposure category. To account for the observed impact of self-reported occupation on urinary Cd, we further adjusted for occupational Cd exposure categories to calculate the geometric mean levels of urinary Cd by race/ethnicity. The differences across populations were similar to that without adjustment for occupational Cd exposure categories but were slightly attenuated in Latinos (Table 3). The highest geometric mean level of urinary Cd was observed in Latinos (0.864 ng/mL), followed by Native Hawaiians (0.836 ng/mL), Japanese Americans (0.813 ng/mL), African Americans (0.806 ng/mL) and lowest in Whites (0.743 ng/mL; Table 3). Additionally, Latinos had the highest level of urinary Cd across all occupational categories (Figure 3). There was no evidence of an interaction between occupational Cd exposure categories and race/ethnicity on urinary Cd levels in any of the analyses (*p* = 0.240).

## 4. Discussion

In this cross-sectional study of current smokers from MEC that contributed a biospecimen, we observed significantly different urinary Cd levels across racial/ethnic groups, even after adjusting for internal smoking dose (TNE) and smoking duration. Latino smokers had the highest geometric mean urinary Cd levels followed by Native Hawaiian, Japanese American, African American, and White smokers. We also observed differences in urinary Cd levels based on a broad classification of the likelihood of occupational Cd exposure after adjusting for smoking measures and racial/ethnic groups. To our knowledge, this is the first study to examine the racial/ethnic differences in urinary Cd among smokers with differential risk for lung cancer.

Inter-individual variation in Cd exposure is well-documented. For example, prior studies using population-level data from The National Health and Nutrition Examination Survey (NHANES) have shown a wide range of urinary Cd levels across individuals and have consistently found that levels of this biomarker were higher in females compared to males and differed by race/ethnicity with differences seen across survey years [16,17,20,21,27,28,29]. Our findings are consistent with these reports as we found that after accounting for race/ethnicity, age, creatinine, and measures of smoking, females had higher urinary Cd levels compared to males. Levels also differed by race/ethnicity with Latino smokers having the highest level and White smokers having the lowest level of urinary Cd in our study of smokers. Differences in intrinsic factors, such as differences in absorption, metabolism, and/or excretion rates could potentially be responsible for such findings [2,30,31,32]. However, differences in exposure to Cd sources other than smoking should also be considered.

Cadmium is a well-known environmental and industrial pollutant present in occupations such as repair service, metal, mining, and transportation services [20,21]. Despite the implementation of occupational standards and guidelines for permissible Cd exposure limits, workers in a wide variety of occupations are still potentially at risk for Cd exposure [33]. Prior studies have reported higher levels of urinary Cd in participants who worked in occupations and industries, such as vehicle mechanics, transportation, construction, repair service, mining, and metal industry, and that the proportion of workers within these occupations varied by racial/ethnic group [20,21]. Therefore, we further investigated the potential contribution of environmental exposure to Cd from occupational and industrial sources of Cd exposure, using a broad measure of occupational Cd exposure. Indeed, smokers categorized in the ‘Likely exposed’ and ‘Possibly exposed’ occupational Cd exposure categories self-reported working as laborers, factory workers, craftsperson or small business owners in the automotive repair, metal production and processing, mining, quarrying, rock crushing or cement manufacturing industry, and other occupations and industries previously associated with Cd exposures in workers [8,11,21,26]. We also found the relative proportion of smokers by racial/ethnic groups differed significantly across our broad category of occupational Cd exposure, particularly Latinos and Native Hawaiians made up the majority of the “Likely exposed” and “Possibly exposed” categories which suggests occupation may in part explain higher levels of urinary Cd in these two racial/ethnic groups. However, after adjustment for occupational categories (in addition to adjustment for smoking dose and duration), urinary Cd remained elevated in Latino and Native Hawaiian smokers. This is likely due to the relatively crude nature of occupational Cd exposure measures available in our study, as the occupational data collection in MEC was not designed to specifically identify potential occupational exposure to Cd. Nevertheless, our findings indicate that occupation is a potentially important contributor to Cd exposure in our study population, beyond measures of smoking, and should be investigated in other racially/ethnically diverse studies.

It is important to note that the racial/ethnic differences in urinary Cd reported here are not consistent with other studies of tobacco biomarkers and patterns of lung cancer risk found in this population of smokers. The majority of tobacco-specific and tobacco-related biomarkers of exposure analyzed in previous MEC studies agreed with the direction of lung cancer risk in the MEC cohort, with the highest levels being found among African American smokers, intermediate among White smokers, and lowest among Japanese American smokers [1,4,7,22,34]. Only one biomarker measured in these smokers shows trends by race/ethnicity that are similar to urinary Cd and that is urinary phenanthrene tetraol (Phe-T), a biomarker of the polycyclic aromatic hydrocarbon (PAH) phenanthrene and a surrogate measure of PAH exposure. After adjustment for age, sex, BMI, and TNE, urinary Phe-T levels were highest in Latinos followed by African Americans, intermediate levels in Japanese Americans, and lowest levels in Whites [6]. Major sources of exposure to PAH are similar if not the same sources of exposure to Cd; besides smoking, sources of exposure to PAH include environmental, occupational exposures (e.g., aluminum and coke production industry, paving and roofing using coal tar, etc.), and air pollution [35]. Furthermore, Latinos in California (particularly the greater Los Angeles area where our population was recruited) were reported to be disproportionally exposed to environmental health hazards [34]. Collectively, these reports and our findings suggest that environmental exposures could be an important factor contributing to urinary Cd, Phe-T, and potentially other biomarkers in MEC smokers. The lower risk of lung cancer observed in Latinos, despite the higher levels of urinary Cd and Phe-T, warrants further research that may shed light on certain metabolic and/or genetic factors involved. For example, some studies suggest epigenetic mechanisms are involved in the process between exposure to environmental heavy metals, including Cd, and cancer susceptibility [36,37]. However, no single mechanism has yet to be identified. Epigenetic studies in our population of smokers are underway to assess this association.

The main strength of this study is the use of a well-characterized multi-ethnic cohort of current smokers that have extensive epidemiologic data and measures of tobacco constituent biomarkers. Notably, many studies lack a level of detail to account for contributions from smoking and very few use a measure of urinary Cd to investigate long-term exposure. Unlike such previous studies, the current study quantified Cd content in biological samples of current smokers with better measures of smoking intensity (TNE) [2]. TNE is an excellent biomarker for nicotine uptake and total tobacco exposure as it accounts for about 85% of the internal nicotine dose and is reflective of total smoke intake. This measurement allowed us to accurately assess individual differences from cigarette smoke and identify inter-individual differences in urinary Cd that are independent of the amount of smoking [38,39,40].

The results of this study should be interpreted with the following limitations in mind. First, urinary Cd was assessed in samples that were collected by two different methods, overnight urine samples (participants recruited in Hawaii) or first morning urine samples (participants recruited in California), which can lead to variation in biomarker concentration due to differences in water content of urine. To account for these differences and the potential impact on urine dilution, we adjusted for urine creatinine in the statistical model [41]. It should also be noted that the overall geometric mean urinary Cd levels reported in our study are almost 2-fold higher in comparison to those reported for cigarette smokers in earlier studies [17,20,21]. These differences are likely attributable to our study population being older, with an overall median age of 64 years (range: 46–87 years), whereas other studies incorporate a wider range of ages (e.g., 6 to 70+ years). Literature has consistently shown that urinary Cd increases with age which is the likely explanation of the overall higher levels observed in this study [16,20,21,29]. Second, we lacked detailed information for each occupation or industry reported within our study, which may have led to misclassification of occupational Cd exposure in some participants. However, individuals in the ‘Likely exposed’ and ‘Possibly exposed’ occupational Cd exposure category had significantly higher urinary Cd levels than the ‘Not Likely exposed’ category, and this association remained consistent across all five race/ethnicity groups adding strength to our approach and observations. Third, diet and drinking water may also contribute to Cd exposure [8]. In our study, dietary recall was assessed by a food frequency questionnaire (FFQ) on average 10 years before biospecimen collection and may not accurately reflect current dietary intake at the time of urine collection, and therefore was not included in our analysis. However, the contribution of Cd from the diet is expected to be minimal compared to smoking and occupational exposures and is not likely to play a meaningful role in lung cancer risk where inhalation exposure routes are more relevant. Lastly, as noted earlier, environmental exposures (e.g., air pollution, toxic chemical releases, etc.) are likely to be important contributors to inter-individual variation in urinary Cd across smokers, but such exposures were not accounted for in this study [35].

## 5. Conclusions

In conclusion, the findings of this study demonstrate that independent of smoking, urinary Cd levels in MEC current smokers at the time of biospecimen collection differed by race/ethnicity, was higher in females, and was higher in smokers with the likelihood of occupational Cd exposure. While racial/ethnic trends in urinary Cd were not consistent with previously reported differences in lung cancer risk, this study is an important contribution to the overall characterization of exposure to cigarette smoke constituents among MEC smokers and could be helpful in future studies of individual characteristics that are associated with a lower risk for cancer despite higher carcinogenic exposures. Future research is needed to identify additional factors including environmental, genetic and the role of smoking dependence contributing to urinary Cd levels in smokers to better understand the role of Cd exposure in the observed racial/ethnic differences in lung cancer risk.

## Figures and Tables

**Table 1:** Main characteristics of Multiethnic Cohort Study participants overall and by race/ethnicity (N = 1956).

	Overall	African Americans	Native Hawaiians	Whites	Latinos	Japanese Americans
	N = 1956	N = 280	N = 294	N = 387	N = 411	N = 584
**N (%)**						
Sex						
Males	925 (47.3)	87 (31.1)	109 (37.1)	170 (43.9)	217 (52.8)	342 (58.6)
Females	1031 (52.7)	193 (68.9)	185 (62.9)	217 (56.1)	194 (47.2)	242 (41.4)
Education						
≤12th grade	785 (40.1)	108 (38.6)	164 (55.8)	90 (23.3)	260 (63.3)	163 (27.9)
Vocat./some college	681 (34.8)	116 (41.4)	93 (31.6)	122 (31.5)	111 (27.0)	239 (40.9)
≥Graduated college	490 (25.1)	56 (20.0)	37 (12.6)	175 (45.2)	40 (9.7)	182 (31.2)
**Median (interquartile range—25th and 75th percentile)**						
Age, yrs.	63.7 (59.3, 69.5)	64.5 (59.9, 69.1)	61.0 (56.9, 65.9)	62.5 (59.2, 69.3)	65.7 (61.7, 70.8)	63.3 (59.1, 69.8)
Smoking duration, yrs.	43.5 (34.5, 46.5)	37.5 (34.5, 46.5)	37.5 (33.5, 46.5)	44.5 (35.5, 46.5)	43.5 (34.5, 48.0)	43.5 (35.5, 46.5)
Average CPD	10 (6, 20)	10 (5, 18)	15 (9, 20)	20 (10, 20)	8 (4, 12)	12 (10, 20)
Urinary TNE, nmol/mL	32.4 (19.6, 52.8)	44.5 (28.3, 70.7)	30.3 (19.3, 46.3)	35.7 (21.9, 57.2)	32.7 (20.6, 54.0)	27.4 (15.7, 42.9)
Urinary creatinine, mg/dL	76.9 (44.8, 127.9)	113.4 (66.7, 167.8)	74.6 (40.0, 115.8)	62.7 (39.1, 109.1)	92.0 (56.6, 141.8)	67.6 (39.3, 113.0)

CPD—self-reported cigarettes per day; TNE—total nicotine equivalents.

**Table 2:** Geometric mean (95% CI) of urinary cadmium concentration (ng/mL) by sex, race/ethnicity and occupational cadmium exposure categories among MEC current smokers.

		Model 1 ^a^		Model 2 ^b^	
	N	Geometric Mean (95% CI)	*p*	Geometric Mean (95% CI)	*p*
**Overall**	1956	0.802 (0.778, 0.827)		0.813 (0.789, 0.838)	
**Sex**					
Male	925	0.736 (0.707, 0.765)	Ref.	0.743 (0.714, 0.772)	Ref.
Female	1031	0.880 (0.842, 0.917)	<0.001	0.898 (0.860, 0.936)	<0.001
**Race/ethnicity**					
African American	280	0.821 (0.764, 0.878)	0.127	0.807 (0.753, 0.861)	0.044
Native Hawaiian	294	0.815 (0.757, 0.872)	0.167	0.836 (0.779, 0.893)	0.005
White	387	0.764 (0.715, 0.812)	Ref.	0.736 (0.691, 0.781)	Ref.
Latino	411	0.834 (0.785, 0.883)	0.044	0.871 (0.821, 0.922)	<0.001
Japanese American	5884	0.781 (0.740, 0.822)	0.566	0.811 (0.769, 0.853)	0.009
**Occupational Cd exposure**					
Likely exposed	149	0.924 (0.833, 1.014)	0.002	0.891 (0.808, 0.975)	0.029
Possibly exposed	343	0.847 (0.791, 0.902)	0.034	0.856 (0.802, 0.911)	0.047
Not likely exposed	1106	0.779 (0.748, 0.810)	Ref.	0.795 (0.763, 0.827)	Ref.
Unknown exposure	358	0.775 (0.725, 0.824)	0.885	0.788 (0.739, 0.837)	0.806

^a^ Model 1: adjusted for self-reported race/ethnicity (for overall, sex, and occupational Cd exposure specific models), sex (for overall, race/ethnicity, and occupational Cd exposure specific models), age at urine collection, creatinine (natural log), and maximum education attainment level. ^b^ Model 2: Model 1 further adjusted for urinary total nicotine equivalents (TNE) and smoking duration (years). *p* when compared to referent.

**Table 3:** Geometric mean (95% CI) of urinary cadmium concentration (ng/mL) by sex, race/ethnicity and occupational cadmium exposure categories among MEC current smokers.

		Model 3 ^a^	
	N	Geometric Mean (95% CI)	*p*
**Overall**	1956	0.802 (0.778, 0.827)	
**Sex**			
Male	925	0.736 (0.707, 0.765)	Ref.
Female	1031	0.880 (0.842, 0.917)	<0.001
**Race/ethnicity**			
African American	280	0.821 (0.764, 0.878)	0.127
Native Hawaiian	294	0.815 (0.757, 0.872)	0.167
White	387	0.764 (0.715, 0.812)	Ref.
Latino	411	0.834 (0.785, 0.883)	0.044
Japanese American	5884	0.781 (0.740, 0.822)	0.566

^a^ Model 3: Model 2 (adjusted for self-reported race/ethnicity [for overall and sex specific models], sex (for overall and race/ethnicity specific models], age at urine collection, creatinine (natural log), maximum education attainment level, urinary total nicotine equivalents (TNE) and smoking duration [years]) further adjusted for occupational cadmium exposure categories. *p* when compared to referent.

## Data Availability

Restrictions apply to the availability of these data. Data was obtained from via an approved proposal by the Multiethnic Cohort Study Research Committee. Data requests should be made to the Multiethnic Cohort (MEC) study (see “Data Sharing” on the MEC website: https://www.uhcancercenter.org/mec). Investigators need to submit a formal application that will be evaluated internally by the MEC Research Committee before any data are released. Documentation of IRB approval is required for all projects requesting to use MEC data.

## Supplementary Materials

The following are available online at https://www.mdpi.com/1660-4601/18/5/2669/s1. Table S1: Categorization of Multiethnic Cohort (MEC) current smokers into occupational cadmium (Cd) exposure categories based on their combined response to two questions regarding longest history of industry and occupation worked. Table S2. Median and interquartile range of urinary cadmium (Cd) concentrations (ng/mL) overall and stratified by sex and race/ethnicity. Table S3. Geometric mean (95% CI) of urinary cadmium (Cd) concentration (ng/mL) by sex and race/ethnicity among Multiethnic Cohort (MEC) current smokers—this analyses omits creatinine from the model. Table S4. Geometric mean (95% CI) of urinary cadmium (Cd) concentration (ng/mL) by sex and race/ethnicity among Multiethnic Cohort (MEC) current smokers—this analyses compares two different modeling techniques for smoking measures.
