# Supplementary material for: Ethnic Differences of Urinary Cadmium in Cigarette Smokers from the Multiethnic Cohort Study
Source: Int J Environ Res Public Health. 2021 Mar 6;18(5):2669. doi: 10.3390/ijerph18052669 (PMC7967490; doi:10.3390/ijerph18052669)
Supplement: Supplementary file 1 [file ijerph-18-02669-s001.pdf]

International Journal of
Environmental Research and Public Health

MDPI

Supplementary Materials

Ethnic differences of urinary cadmium in cigarette smokers from the Multiethnic Cohort Study

Shannon S. Cigan [1,2*], Sharon E. Murphy [3,4], Bruce H. Alexander [2,5], Daniel O. Stram [6], Dorothy K. Hatsukami [4,7], Loic Le Marchand [8], Sungshim L. Park [8†], Irina Stepanov [2,4†]

Table S1: Categorization of Multiethnic Cohort (MEC) current smokers into occupational cadmium (Cd) exposure categories based on their combined response to two questions regarding longest history of industry and occupation worked.

Exposure Category	Industry Indicator [a]	Occupational Indicator [b]
Likely exposed [c]	Yes	Yes
Possibly exposed	Yes	No
Possibly exposed	Yes	Unknown
Possibly exposed	No	Yes
Possibly exposed	Unknown	Yes
Not likely exposed	No	No
Not likely exposed	No	Unknown
Not likely exposed	Unknown	No
Unknown exposure	Unknown	Unknown

[a] 'Yes' indicators include the following industry types from the MEC baseline questionnaire that would likely have Cd exposure: 'metal production or processing, mining, quarrying, rock crushing, or cement manufacturing, plastic production or processing, gasoline refining or distribution, rubber or tire manufacturing, shipyard work, farming, furniture making or woodworking, automotive repair, pesticide production and paint production or use'.

[b] 'Yes' indicators include the following occupation types from the MEC baseline questionnaire that would likely have Cd exposure: 'laborer or farm worker, factory worker or machine operator, and craftsperson'

[c] exception for participants who indicated they were 'small business owner' - if a small business owner worked in an industry that is known to be exposed to Cd [a], participants were grouped in the 'Likely exposed' category.

International Journal of
Environmental Research and Public Health

MDPI

Table S2. Median and interquartile range of urinary cadmium (Cd) concentrations (ng/mL) overall and stratified by sex and race/ethnicity.

	N	Median (Interquartile Range: 25th, 75th)	*P*
**Overall**	1,956	0.60 (0.36, 1.02)	
**Sex**			
**Male**	925	0.66 (0.36, 1.02)	Ref.
**Female**	1031	0.54 (0.36, 0.96)	0.004
**Race/ethnicity**			
**African American**	280	0.84 (0.48, 1.29)	<0.001
**Native Hawaiian**	294	0.60 (0.36, 0.96)	0.013
**White**	387	0.48 (0.30, 0.78)	Ref.
**Latino**	411	0.72 (0.42, 1.08)	<0.001
**Japanese American**	584	0.54 (0.30, 0.84)	0.424

P value when compared to referent using Wilcoxon-Mann-Whitney test

International Journal of
Environmental Research and Public Health

MDPI

Table S3. Geometric mean (95% CI) of urinary cadmium (Cd) concentration (ng/mL) by sex and race/ethnicity among Multiethnic Cohort (MEC) current smokers – this analyses omits creatinine from the model.

		Model 1 [a]		Model 2 [b]	
	N	Geometric mean (95% CI)	*P*	Geometric mean (95% CI)	*P*
Overall	1,956	0.807(0.777,0.838)		0.836 (0.804,0.867)	
Sex					
Male	925	0.859 (0.814,0.905)	Ref.	0.831 (0.792,0.870)	Ref.
Female	1,031	0.763 (0.725,0.800)	<0.001	0.840 (0.800,0.880)	0.725
Race/ethnicity					
African American	280	1.085 (0.986,1.184)	<0.001	0.922 (0.850,0.993)	<0.001
Native Hawaiian	294	0.778 (0.708,0.848)	0.029	0.844 (0.777,0.911)	<0.001
White	387	0.681 (0.628,0.735)	Ref.	0.671 (0.625,0.718)	Ref.
Latino	411	0.924 (0.853,0.995)	<0.000	0.960 (0.894,1.025)	<0.001
Japanese American	5884	0.692 (0.648,0.736)	0.758	0.796 (0.748,0.845)	<0.001

[a] Model 1: adjusted for self-reported race/ethnicity (for overall and sex specific models), sex (for overall and race/ethnicity specific models), age at urine collection, and maximum education attainment level.

[b] Model 2: Model 1 further adjusted for urinary total nicotine equivalents (TNE) and smoking duration (years)

P when compared to referent

International Journal of
Environmental Research and Public Health

MDPI

Table S4. Geometric mean (95% CI) of urinary cadmium (Cd) concentration (ng/mL) by sex and race/ethnicity among Multiethnic Cohort (MEC) current smokers – this analyses compares two different modeling techniques for smoking measures.

		**Model 1** [a]		**Model 2** [b]	
	**N**	**Geometric mean (95% CI)**	***P***	**Geometric mean (95% CI)**	***P***
Overall	1,956	0.803 (0.779, 0.826)		0.814 (0.789, 0.839)	
Sex					
Male	925	0.737 (0.708, 0.765)	Ref.	0.744 (0.715, 0.773)	Ref.
Female	1,031	0.880 (0.844, 0.916)	<0.001	0.898 (0.860, 0.936)	<0.001
Race/ethnicity					
African American	280	0.801 (0.748, 0.854)	0.027	0.812 (0.757, 0.866)	0.044
Native Hawaiian	294	0.819 (0.764, 0.874)	0.006	0.837 (0.780, 0.895)	0.006
White	387	0.724 (0.680, 0.768)	Ref.	0.740 (0.695, 0.785)	Ref.
Latino	411	0.868 (0.818, 0.917)	<0.001	0.864 (0.814, 0.914)	0.014
Japanese American	5884	0.795 (0.755, 0.836)	0.011	0.811 (0.769, 0.853)	<0.001

[a] Model 1: adjusted for self-reported race/ethnicity (for overall and sex specific models), sex (for overall and race/ethnicity specific models), age at urine collection, maximum education attainment level, total nicotine equivalents (TNE; natural log), and smoking duration (years).

[b] Model 2: adjusted for self-reported race/ethnicity (for overall and sex specific model), sex (for overall and race/ethnicity specific model), age at urine collection, creatinine (natural log), maximum education attainment level, and cumulative total nicotine equivalents (TNE*smoking duration).

P when compared to referent.
